# From Experiments to AI: A Comparative Review of Machine Learning Approaches for Predicting Nanofluid Thermophysical Properties

**DOI:** 10.3390/nano16040272

**Published:** 2026-02-20

**Authors:** Salim Al. Jadidi, Rekha Moolya, Rajendra Padidhapu, Sivasubramanian Subramanian, Shivananda Moolya

**Affiliations:** 1 Department of Engineering, College of Engineering and Technology, University of Technology and Applied Sciences, Muscat 133, Oman; salim.aljadidi@utas.edu.om (S.A.J.); siva.subramanian@utasoman.onmicrosoft.com (S.S.); 2 VTU(RC)CMR Institute of Technology, Bengaluru, Visvesvaraya Technological University, Belagavi 590018, India; rekha.moolya_maths@gopalancolleges.com (R.M.); rajendra.p@cmrit.ac.in (R.P.); 3 Department of Mathematics, Gopalan College of Engineering and Management, Bengaluru, Visvesvaraya Technological University, Belagavi 590018, India

**Keywords:** nanofluids, viscosity, machine learning, thermophysical property

## Abstract

The applications of nanofluids are widely beneficial in heat transmission and cooling systems. Nanofluid viscosity and thermal conductivity have a substantial effect on heat transfer applications and on devices such as solar and geothermal systems. Machine learning models enable faster, less expensive modeling of nanofluid thermophysical properties. These models are secure for future studies and in the development of nanotechnology. In this review, shape, size, temperature, and volume concentration are considered as inputs to develop several machine learning methods, such as artificial neural networks, support vector regression, decision trees, and random forests. These models were analyzed by comparing their R^2^ values, and the results indicated that machine learning-based models generally exhibited more reliable performance than the other approaches. The observation in this review was that thermal conductivity increases with temperature and volume fractions, whereas viscosity decreases with size, temperature, and volume fractions. To determine the optimal nanoparticle type, size, and concentration for specific applications such as data center cooling and high-heat-flux electronics, future research may employ ML-based optimization techniques.

## 1. Introduction

Nanotechnology is applied to improve the efficiency of cooling and heating systems across various scientific domains. Nanofluids are two-phase systems composed of a base fluid and nanoparticles. The fundamental fluids are generally biological liquids, ethylene glycol, water, and oil. Nanoparticles are incredibly tiny particles. Particle sizes typically range from 1 to 100 nm. Metals, oxides, carbides, or carbon nanotubes are the materials utilized to make nanoparticles for use in nanofluids. Compared with the base fluid, these particles exhibit improved thermal conductivity and a higher convective heat transfer coefficient. Basic fluids like oil and ethylene propylene glycol are crucial to many engineering specialties, including mechanical, thermal energy, heating and cooling systems, and biomedical fields. Because of their improved thermophysical properties, such as thermal conductivity, viscosity, density, and specific heat capacity, nanofluids, engineered suspensions of nanoparticles in traditional base fluids, have become viable heat transfer media. Heat exchangers, energy storage, electronics cooling, automotive systems, and data center cooling technologies are just a few of the many thermal management systems that have benefited from these exceptional qualities. Therefore, an accurate understanding of the thermophysical characteristics of nanofluids is crucial for numerical modeling, performance optimization, and dependable system design. Nanofluid characteristics remain difficult to determine experimentally, despite their significance. Measurements are frequently costly, time-consuming, and sensitive to a wide range of variables, such as temperature, base fluid type, preparation technique, nanoparticle material, size, shape, and concentration. Furthermore, inconsistent patterns and dispersed datasets are sometimes the result of experimental inconsistencies between several studies. Although helpful, classical theoretical and semi-empirical correlations often rely on restrictive assumptions and cannot be generalized across a wide range of nanofluid systems, especially under different thermophysical and operational conditions. These drawbacks emphasize the need for more adaptable and reliable prediction methods. Abbas et al. [1] studied the performance of cobalt oxide nanoparticles in different applications. They found that the manufactured nanoparticles are suitable for electrode material and as thermal sensors. These days, sophisticated machine learning is used to predict these properties, leading to the development of numerous models. These techniques are more effective, quicker, more precise, and more useful than the experiments by Ahmadloo and Aziz [2]. Currently, artificial neural networks (ANNs) are widely used in many thermal applications, including heat transfer, flow behavior prediction, and thermophysical property prediction. ANN is a branch of artificial intelligence that draws inspiration from the brain’s natural ability to detect patterns. By applying material informatics to research on functional materials, Shi et al. [3] developed an ANN model that predicts the thermal conductivity, viscosity, and specific heat characteristics of magnetic nanofluids based on carbon using experimental data, yielding a higher statistical coefficient and a lower statistical error index. This result indicated that the need for many parameters and the lack of parameter selection techniques are the main drawbacks of ANN applications. To forecast the densities of nitride nanofluids, Sahaluddin et al. [4] developed a support vector regression (SVR) model that included variables such as temperature, mass percentage, nanoparticle diameter, and molecular weight. In this study, the density of the aluminum nitride–ethylene glycol nanofluid was predicted at 298.150 K and 308.150 K, and nanoparticle sizes of 20 nm and 50 nm and volume fractions of 0.000% and 0.05% were achieved. For the titanium nitride–ethylene glycol nanofluid, the selected input variables included temperatures of 288.150 K and 298.150 K, nanoparticle sizes of 20 nm and 50 nm, and volume fractions of 0.0005%, 0.010%, 0.025%, and 0.050%. In the case of the silicon nitride–ethylene glycol nanofluid, the model inputs consisted of temperatures of 288.150 K, 298.150 K, and 308.150 K, volume fractions ranging from 0.000% to 0.050%, and nanoparticle sizes of 20 nm and 80 nm. In comparison with the Pak and Cho model, higher accuracy is observed at 298.15 K, but deviations occur at other temperatures, which represent a key limitation of their formulation. In contrast, the proposed SVR model does not explicitly rely on temperature and can predict nanofluid density with high accuracy across a range of temperatures. Consequently, the machine learning-based models demonstrate superior predictive performance, indicating improved precision. By forecasting the viscosity and thermal conductivity of nanofluids, Li et al. [5] created an ANN model to evaluate nanofluid stability and demonstrated that the model captures the proposed correlations. This result also indicated that increasing the temperature from 25 to 60 °C enhances thermal conductivity by 9% to 16% at a mass fraction of 2.0 wt%. In contrast, at the same mass fraction and a temperature of 60 °C, an increase of 18.2% in viscosity was observed for nanoparticles with a size of 20 nm. A neural network technique was published by Ekene Jude Onyiriuka [6] to forecast the accuracy of nanofluid heat transfer coefficients. The algorithm is evaluated using R^2^, Mean Absolute Error (MAE), and Root Mean Squared Error (RMSE). The developed models were found to be accurate across all Reynolds numbers (60–59,300), all particle sizes (20–150 nm), and volume fractions ranging from 2% to 3.5%. This study suggests that the mixture model and the Single-Phase Model (SPM) can be further improved by incorporating more accurate nanofluid thermophysical properties into the modeling framework. Ningbo Zhao and Zhiming Li [7] built a radial basis function (RBF) neural network to forecast the thermal conductivity and viscosity of alumina water nanofluids. In this study, they first prepared Al_2_O_3_-H_2_O nanofluids using a two-step method and then developed a radial basis function (RBF) neural network to predict thermal conductivity and viscosity. The network took as inputs various volume fractions, 1.31%, 2.72%, 4.25%, and 5.92%, a nanoparticle size of 30 nm and temperatures from 296 to 313 K and analyzed them using mean absolute percentage error (MAPE), and also showed that it could effectively extract the influences of nanoparticle volume fraction and temperature on the Al_2_O_3_-H_2_O nanofluid’s thermal conductivity and viscosity. Using the backpropagation Levenberg–Marquardt algorithm, Sadeghzaden et al. [8] developed an ANN model to predict the thermal conductivity of TiO_2_ and Al_2_O_3_-H_2_O and compared the results using correlation coefficient values. In this work, TiO_2_–Al_2_O_3_ hybrid nanoparticles were synthesized via the sol–gel technique, considering Al_2_O_3_ compositions ranging from 10% to 60% and temperatures between 300 and 400 K. The findings indicated that a nanofluid concentration of 1.5 vol.% improved thermal conductivity by up to 25%. Additionally, the heat transfer coefficient increased linearly with nanoparticle loading, while its temperature dependence showed a nonlinear trend. To forecast the thermal conductivity and dynamic viscosity of ferromagnetic nanofluid, Mohammad Hemmat Esfe et al. [9] developed an ANN model using 72 experimental data points, with temperature varying from 26 °C to 55 °C, volume fractions between 0.125% and 3.0%, and particle sizes of 40, 70 and 100 nm, demonstrating errors of 2.5% and 2%, respectively, between predicted and experimental correlations. This study indicates that the proposed models are in good agreement with the experimental results. By comparing RMSE values, Durgam and Kadam [10] developed linear regression and ANN models to investigate the thermal conductivity and viscosity of various nanofluids using 473 and 443 experimental data points, respectively, and found them to be more accurate than analytical models. Komeilibirjandi et al. [11] proposed a group-based data management approach and mathematical correlations to predict the thermal conductivity of CuO nanoparticles, and compared the results using R^2^ values and Relative Root Mean Square Error (RRMSE). Using neural networks and correlations, Hemmat Esfe et al. [12] examined the thermal conductivity of Mg(OH)_2_ nanofluids experimentally over volume fractions ranging from 0.001 to 0.02% and temperatures of 24, 35, 45, and 55 °C. They compared the results using mean squared error (MSE), Mean Absolute Error (MAE), and Sum of Squared Errors (SSE). The findings demonstrate that the developed models provide a practical alternative to expensive and time-consuming experimental methods. Hemmat Esfe et al. [13] developed a model to predict the thermal conductivity of Al_2_O_3_/H_2_O nanofluids as a function of solid volume fraction (0.0025, 0.005, 0.01, 0.02, 0.03, 0.04 and 0.05%) and temperature (26, 35, 45, and 55 °C), and 28 experimental data points were used to establish correlation pattern, which was evaluated by using the mean squared error (MSE) and the correlation coefficient. Kumar and Kavitha [14] employed a Gaussian Process Regression model to estimate the thermal conductivity and dynamic viscosity of Al_2_O_3_–water nanofluids, using temperature (293 K to 324 K), particle size (11 nm to 108 nm), and volume fraction (0.01 to 0.05%) as the input parameters, and the results were assessed using the coefficient of correlation (R^2^ value) and Root Mean Square (RMS), and they found that the temperature is the crucial factor to enhance the thermal conductivity ratio. Hojjat et al. [15] synthesized CuO, Al_2_O_3_, and TiO_2_ nanoparticles dispersed in 0.5 wt% Carboxymethyl Cellulose (CMC) and investigated the thermal conductivity of non-Newtonian nanofluids through both experimental analysis and neural network modeling. The study considered particle sizes of 25 nm for Al_2_O_3_, 10 nm for TiO_2_, and 30–50 nm for Cuo, with volume fractions ranging from 0.1% to 4.0% and temperature between 5 and 45 °C as the input parameters. Kurt et al. [16] developed an ANN model to forecast the thermal conductivity of EG-H_2_O solutions. Their regression-based analysis used temperature data between 10 and 80 °C, volume fractions ranging from 0 to 100%, and densities of 974.08–1138.25 kg/m^3^, and it was evaluated using mean absolute percentage error (MAPE) and R^2^ values. This study enables application engineers to estimate the thermal conductivity of ethylene glycol–water solutions without extensive experimental testing, thereby reducing both cost and time. Shateri et al. [17] employed an ANN to estimate the relative viscosity of nanofluids using temperature, base fluid viscosity, nanoparticle volume fraction, particle size, and density as input variables. The model was trained on 3144 experimental data points from available resources, and its physical reliability was validated by verifying that the predicted viscosity followed the expected trends with respect to variations in nanoparticle volume fraction. Esfe et al. [18] created an ANN that predicted the relative viscosity of a nanofluid and used experimental data to forecast the rheological behavior of aluminum oxide–multiwalled carbon nanotubes/5W50 hybrid nano lubricant. The ANN was assessed using statistical and graphical methods. To forecast nanofluid viscosity, Heidari et al. [19] created a feedforward backpropagation multilayer perceptron using temperature (35–71.2 °C), nanoparticle size (7–190 nm), nanoparticle volume fraction (0–9%), nanoparticle density (2650–6310 kg/m^3^**)**, base fluid viscosity (0.394–452.599 cp), and nanofluid relative viscosity (0.56099–9.77636) as input parameters, and evaluated it with the highest average relative deviation and average absolute relative deviation. Ahamadi et al. [20] created multiple machine learning models to predict the viscosity of copper oxide–water nanofluids, using temperature (10–66 °C), nanoparticle size (11–29 nm), and concentration (0–9%) as the input parameters, which were then compared using Average Absolute Percentage Relative Error and R^2^ value. By comparing mean absolute percentage errors, Longo et al. [21] reported that the ANN model showed high accuracy in forecasting the dynamic viscosity of oxide nanoparticles in EG and water by considering particle size (30 ± 10 nm), volume fraction (1, 2 and 4%) and temperature (1–40 °C) for the Al_2_O_3_–water nanofluid with 30 data points and particle size (50 ± 10 nm), volume fraction (1, 2 and 4%) and temperature (1–40 °C) for the Ti_2_O_2_–water nanofluid with 40 data points, respectively. Elsheikh et al. [22] introduced an artificial neural network technique and developed a model to predict the thermal conductivity of copper, aluminum, and titanium oxide with a base fluid. This model was evaluated using Mean Absolute Percent Error and mean squared error, and it can also be tested on datasets with unknown parameter values. Vaferi [23] developed an ANN model based on experimental data covering temperature (1–133.8 °C), particle size (8–283 nm), and volume fraction (0.0013–0.16%). The model was validated against experimental and empirical correlations, and its performance was evaluated using MSE, and the coefficient of determination (R^2^ value). To assess the thermal conductivity of CuO, Al_2_O_3_, and titanium oxide nanofluids with ethylene glycol and compare it with the R^2^ value, Yashawantha and Vinod [24] developed an artificial neural network that can be utilized for a wide range of data. To forecast the relative thermophysical properties of multiwalled carbon nanotube (MWCNT) nanofluids, Bakthavatchalam et al. [25] proposed an ANN approach, which was subsequently validated using experimental data and evaluated using an R^2^ value. Esfe et al. [26] employed the NSGA-II algorithm to optimize temperature and nanoparticle volume fraction to reduce viscosity while enhancing thermal conductivity. They demonstrated that the optimum occurs at a higher temperature. To estimate the viscosity of carbon-based nanomaterials and their hybrid combinations, Olumegbon et al. [27] developed a machine learning model using 120 experimental data points by considering a temperature range from 5 to 100 °C, mass fractions of 0.05, 0.1, 0.2 and 0.5%, viscosity of base fluid of 0.0144–0.8092/Pa s and viscosity of nanofluid ranging from 0.0151 to 1.4740/Pa s and evaluated it using the R^2^ value (correlation coefficient) and RMSE. Mirsaeidi and Yousefi [28] developed ANN models using experimental data, considering volume fraction (0.2–1%) and temperature (10–80 °C) as input variables, and compared them using R^2^ values after conducting experimental research on the density, thermal conductivity, and viscosity of carbon quantum dot nanofluids. To predict the dynamic viscosity of the aluminum oxide–water nanofluid, Kumar and Kavitha [29] built multilayer perceptrons using Gaussian process regression (GPR) and evaluated them using Root Mean Square Error. Based on volume fraction and temperature, He et al. [30] developed an ANN model to predict the thermal conductivity of a hybrid Newtonian nanofluid comprising ZnO, silver, and water, considering various temperatures (25, 30, 35, 40, 45, 50 °C) and volume fractions (0, 0.125, 0.25, 0.5, 1, 1.5, 2%). The results were compared using the correlation coefficient, and this study used a surface-fitting method on the experimental data. To forecast the thermal conductivity of Al_2_O_3_ nanofluids with ethylene glycol and water, Ahmadi et al. [31] used a collective approach to data handling via an ANN. They compared the results using the R^2^ value. To predict the thermal conductivity of the Al_2_O_3_/H_2_O nanofluid according to temperature (10–70 °C), particle size (5–282 nm), and volumetric concentration (0.25–6%), Ahmadi et al. [32] used SVM and an ANN. They compared the results using the correlation coefficient. To forecast multiwalled carbon nanotube–titanium oxide’s thermal conductivity with EG nanofluid, Akhgar et al. [33] created an artificial neural network model by considering temperature (25–50 °C) and volume fractions (0–1%) as input variables. Abidi et al. [34] developed an ANN to evaluate the thermal behavior of a SiO_2_/EG–water nanofluid and compared its performance with MSE and R^2^ values. To estimate the viscosity of nanofluids, Harooni et al. [35] investigated an RBF neural network to estimate the viscosity of several nanofluids, including TiO_2_/DI water, SiO_2_/ethanol, SiO_2_/DI water, CuO/propylene glycol–water, and Al_2_O_3_/water. Their model was trained on 1490 experimental data points, incorporating temperature (238.15–344.35 K), nanoparticle size (7–190 nm), volume fraction (0–9%), nanoparticle density (2.65–6.31 g/cm^3^), and base fluid viscosity (0.394–452.599 cp) as input variables. The predictive performance was assessed using R2, AARD%, and RMSE, and showed that volume fraction has the greatest impact on viscosity, while temperature has the lowest. To determine the specific heat of metallic oxides using EG nanofluids, Alade et al. [36] developed an SVR model using a Bayesian approach using 189 experimental data points by considering temperature (296.31–337.43 K), specific heat capacity of nanoparticle (0.53–0.58 J/K.g), specific heat capacity of ethylene glycol (2.41–2.60 J/K.g) and volume fraction (0.40–8.10%) as input variables. They compared the results using the Relative Average Absolute Deviation (AARD) and the coefficient of correlation, and this was highlighted by comparing them with the analytical models. To predict the thermal conductivity of hybrid nanofluids, Adun et al. [37] created a multilayer perceptron artificial neural network and an SVR model, using 715 experimental data points by considering temperature ranging from 20 to 70 °C, volume fraction 0–3.5%, particle size 1.5–70 nm, mixture ratio 0.15–0.85 and acentric factor of base fluid 0.343–0.659. They compared them using MSE and the correlation coefficient, and the findings confirm that the intelligent model can effectively support scientists and engineers. To estimate the thermal conductivity and dynamic viscosity of Fe_2_O_3_/EG–water, Ahmadi et al. [38] used a genetic algorithm (GA), SVM, and particle swarm optimization–artificial neural networks by considering temperature (20 °C and 50 °C) and volume fraction (0.2% and 0.4%) as input variables. The output was analyzed using R^2^, MSE, and average absolute relative deviation (AARD). The specific heats of titanium and aluminum–silicon with EG were predicted using the SVR model by Alade et al. [39] and compared to the mean absolute percentage error. Moreover, this model uses simple and easy-to-obtain inputs variables such as temperature (287.75–308.43 K), nanoparticle size (20.00–80.00 nm), molecular weight (40.99–140.28 g/mol) and volume fraction (0.000–0.1000%). To estimate nanofluid relative viscosity, Ansari et al. [40] developed a feedforward backpropagation multilayer perceptron (MLP) model by considering temperature, shear rate, nanoparticle size, nanoparticle density, and particle concentration, using 1620 experimental data points. They compared the model using R2 and mean squared error. To estimate the viscosity of titanium nanotubes in EG-H_2_O-based nanofluids, Ali et al. [41] developed a multivariable correlation and an ANN using mass concentration of nanotubes (0 to 1%), temperature (25–65 °C) and shear rate (150–160 s^−1^) as input parameters. They compared them using R^2^ value and AAD% and showed that temperature’s effect on the viscosity is more significant compared to mass fraction. To forecast the thermal conductivity, viscosity, and density of diamond–carboxyl functional groups (COOH)and MWCNT-COOH/water nanofluids, Alrashed et al. [42] created an adaptive neuro-fuzzy inference system and an ANN and compared it with absolute percentage error and Root Mean Square Error. To predict the thermal conductivity and viscosity of graphene platelets and carbon nanotubes (CNTs) in EG–water hybrid nanofluids, Bhanuteja et al. [43] developed machine learning models using concentrations ranging from 0.5, 0.25, 0.125 to 0.0625%, temperatures ranging from 50 to 70 °C, and carbon nanotubes with 3–15 micron length and 30–50 nm diameter, and compared them using R^2^ values. Abushanab et al. [44] developed SVR, an ANN, and ANFIS to predict the viscosity of polyalphaolefin boron nitride nanofluids using 540 experimental data points, and the results were compared with the R^2^ value. This study specifically showed ANNs will be more effective in prediction. To predict the thermal conductivity of Al_2_O_3_- and CuO-based water nanofluids, Singh and Sharma [45] built a machine learning model using temperature (20–50 °C), volume fraction (0.05–3%) and particle size (11, 45, 150 nm) as input parameters. The model was analyzed using MAE, RMSE, and the coefficient of determination, and this approach can be extended to various nanoparticles and base fluids. To forecast the thermal conductivity, viscosity, and specific heat of the ZrO_2_/EG nanofluid, Sundar and Mewada [46] built a multilayer ANN with a perceptron feedforward and backpropagation architecture by using volume fraction (0.2, 0.4, 0.6, 0.8, 1.0%) and temperature (20–60 °C) as input parameters and compared it with root mean square error. In this study, they used a two-step method to prepare the stable nanofluids. Adaptive neural fuzzy inference and a group data handling technique were developed by Fadhal et al. [47] to predict the dynamic viscosity of nanofluids containing MgO nanoparticles using temperature (10–60 °C), volume fraction (0.125–7.2%), and size (20–40 nm) as input parameters and they later compared the results using the R^2^ value. To forecast the viscosity and thermal conductivity of multiwalled carbon nanotube nanofluids, Maqsood et al. [48] developed an ANN and response surface methodology. To forecast the thermal conductivity of the water-EG/MWCNT-Al_2_O_3_ hybrid nanofluid, Fuxi et al. [49] trained the network using the Levenberg–Marquardt algorithm by considering volume fraction (0, 0.001, 0.002, 0.004, 0.008, and 0.0016%) and temperature (25, 30, 35, 40, 45, and 50 °C) as input variables. They compared the results using coefficient correlation and absolute error. To forecast the thermal conductivity of oxide of tungsten (WO)-MWCNT/hybrid engine oil, Soltani et al. [50] built an ANN model using volume fraction (0.05 to 0.6%) and temperature (20–60 °C) as input parameters and compared it based on MSE and Mean Absolute Error. To forecast the viscosity of hybrid nanofluids, Adun et al. [51] constructed an optimizable Gaussian process regression model using input parameters, such as temperature (5–80 °C), volume fraction (0–3%), particle size (4–40 nm), the acentric factor of the base fluid (0.343–0.714) and nanoparticle density (0.25–10.5 g/cm^3^), which was evaluated using R^2^ value and MSE. This study says that there is a need for robust ML models that can predict this property. To forecast silicon, titanium, and aluminum nitride densities in EG solution, Singh et al. [52] built a Gradient Boost Regression model using temperature (288.15–308.15 K), mass fraction (0.00–0.05%), nanoparticle size (20.00–80.00 nm) and molar mass (61.87–140.28) for 72 samples and compared it with Mean Absolute Deviation. To estimate the thermal conductivity of the MWCNT–liquid paraffin nanofluid, Rostami [53] developed a new machine learning model using 28 data points, with mass fraction (0.005–5%) and temperature (25–70 °C) as input parameters, and the model was evaluated using an R^2^ value. In this study, two numerical strategies were used, an optimization-based ANN model and response surface methodology (RSM) on data points. To anticipate the thermal conductivity of several nanofluids with EG, Wang et al. [54] developed an ANN model using 391 experimental data points by considering temperature (4–90 °C), volume concentration (0.05–10%), particle size (2–282 nm) and thermal conductivities of the base fluid and nanoparticles as input variables and evaluated it using mean squared error and the coefficient of determination. To forecast the thermal conductivity and dynamic viscosity of nanofluids, Karimipour et al. [55] created Group Method of Data Handling (GMDH)-type neural networks using particle size (15–30 nm), mass fraction (2.5, 1.5, 0.5, 0.25%), and temperature (25, 40, 55, 70, 100 °C) as input variables. The analysis that followed was for the relative Pr number. To calculate the actual viscosity of Al_2_O_3_, CuO, TiO_2_, and SiO_2_, M. Mehrabi et al. [56] developed an FCM-based adaptive neuro-fuzzy inference system (FCM-ANFIS) using water as the base fluid and volume concentration, temperature, and size of the nanoparticles as the input parameters, with a set of 536 experimental data points. The performance of these models was evaluated using MAE, MRE, and RMSE. Zhu et al. [57] developed several machine learning models to forecast the thermal conductivity of Al_2_O_3_-EG using particle size (12–282 nm), temperature (411.10–322.57 K), and volume fraction (0.00–3.99%) as input parameters and visualized model performance using a Taylor diagram and regression analysis. In this study, the significance of each feature for the prediction was determined using SHAP analysis. Based on the evaluation criteria employed in this work, including MSE, RMSE, MAE, R^2^, and MAPE, the gradient boosting decision tree was found to be the best-performing model. Babu et al. [58] prepared the nanofluid using a volume fraction ranging from 0.01% to 0.1%. Then, they created an artificial neural network (ANN) model to predict the thermal conductivity and viscosity of alumina–water. According to this work, chemical and mechanical engineers can use this technology to select appropriate nanofluids and improve equipment thermal performance. Erdogan et al. [59] developed an artificial neural network (ANN) model with 99% accuracy to predict the viscosity of water-based nanofluids containing Al_2_O_3_, TiO_2_, and ZnO over volume fractions of 0.1% to 1%. Moreover, M.T. Jamal-Abad et al. [60] showed the effective viscosity of Al_2_O_3_. The viscosity of oil containing CuO and TiO_2_ nanoparticles decreased with nanoparticle addition at some volume fractions and shear rates. M. Parvar et al. [61] experimentally demonstrated that thermal conductivity improves with concentration and that dynamic viscosity decreases with increasing temperature for ZnO nanoparticles in transformer oil. Estimating the thermophysical characteristics of nanofluids experimentally is challenging and time consuming. Researchers developed several models to forecast the thermophysical properties of nanofluids. This model’s performance is dependent on several variables, including the algorithms and the input variables selected. Machine learning (ML) methods have drawn significant attention lately as powerful data-driven tools that can predict intricate nonlinear interactions without making explicit physical assumptions. To predict nanofluid thermophysical properties more accurately than traditional correlations, machine learning algorithms such as artificial neural networks, support vector machines, decision trees, ensemble methods, and hybrid models are increasingly used. ML models provide quick predictions, reduced reliance on experiments, and greater flexibility across various nanofluid compositions and operating ranges by leveraging experimental databases. However, significant restrictions remain despite the growing number of ML-based studies in nanofluid research. Current research frequently focuses on specific nanofluid types or restricted parameter ranges, limiting the model’s generalizability. Uncertainty analysis, rigorous validation across separate datasets, and systematic dataset pretreatment are absent from much of the research. Additionally, the interpretability of ML models, which is crucial for physical knowledge and confidence in predictions, is still not adequately addressed, and comparable evaluations across various ML algorithms are sometimes lacking. Therefore, a thorough assessment of the broader applicability and reliability of ML techniques for predicting nanofluid properties is still pending. Inspired by these gaps, this review offers a methodical and critical evaluation of machine learning methods for forecasting the thermophysical properties of nanofluids. By summarizing existing experimental datasets, classifying machine learning techniques, comparing model performance measures, and highlighting important issues and recommended practices, this paper connects the dots between experimental measurements and data-driven intelligence. The literature reviewed in this study was collected from reputed scientific databases, including Scopus, Science Direct, and Google Scholar. The search was conducted using combinations of keywords such as nanofluids, thermal conductivity, viscosity, machine learning, artificial neural networks, radial basis function, support vector regression, particle size, volume fraction, and temperature. The inclusion criteria were limited to peer-reviewed journal articles that investigated the thermophysical properties of nanofluids using experimental data and data-driven or machine learning-based models, and that reported relevant input parameters and model performance metrics. This paper reviews the thermophysical characteristics of nanofluids utilizing machine learning techniques.

## 2. Nanoparticle Types and Their Applications

**Figure 1:**
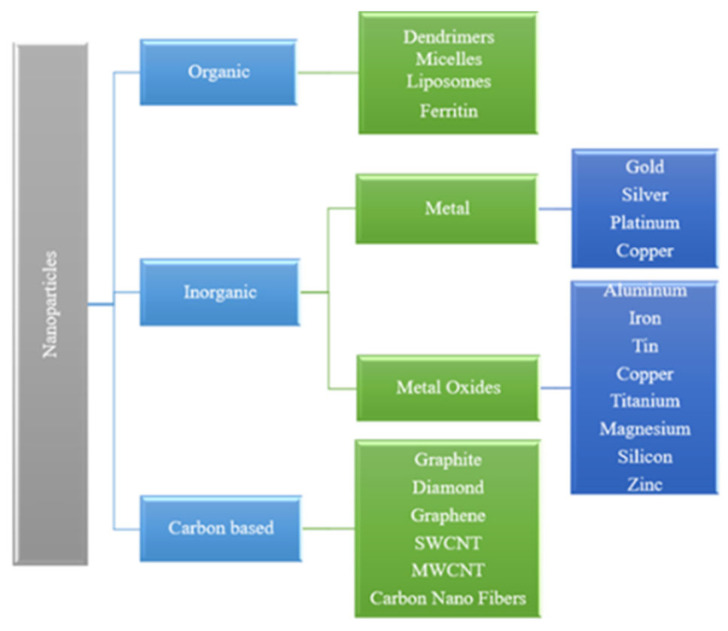
Nanoparticle types.

Nanoparticles are classified into many types based on size, composition, and chemical and physical properties. These nanoparticles are grouped into three types: polymeric, lipid-based, and carbon-based. Artificial nanoparticles, such as metals, quantum dots, nanotubes, sunscreen pigments, and nanocapsules, are well-defined and have an organic coating. Because nanoparticles are so small, their increased surface area enhances heat transfer. The specific heat value of these particles is high. In DNA fingerprinting, magnetic and gold nanoparticles are used as lab tracers to identify cancer stem cells and determine whether DNA is present in a sample [62]. Figure 1 shows the different forms of nanoparticles. The reduction in nanoparticle size in base fluids to a powder or semisolid form thus enhanced particle dispersion. One-step and two-step methods will be used to get these fluids. Solid nanoparticles significantly improve the thermophysical properties of common thermal liquids, such as water, EG, and oil, when dispersed in them. The thermophysical properties of nanofluids play a crucial role in engineering applications, particularly in heating and cooling processes.

## 3. Machine Learning Models

Machine learning involves algorithms designed to solve specific tasks. The performance of these algorithms depends on the amount of input data. Developing models for dynamical systems such as heat transfer, fluid flow, climate dynamics, brain dynamics, and biological systems is complex. Machine learning is concerned with algorithms designed to accomplish a specific task, whose performance improves with additional data. These approaches are used to estimate the thermophysical characteristics of nanofluids. In the present study, various machine learning methods, including artificial neural networks (ANN), support vector machines, and multilayer perceptron methods, are used to predict the thermophysical properties of nanofluids. Based on the reviewed studies, machine learning techniques have demonstrated superior accuracy in predicting nanofluid thermophysical properties, including thermal conductivity, viscosity, and specific heat.

### 3.1. Artificial Neural Networks (ANNs)

**Figure 2:**
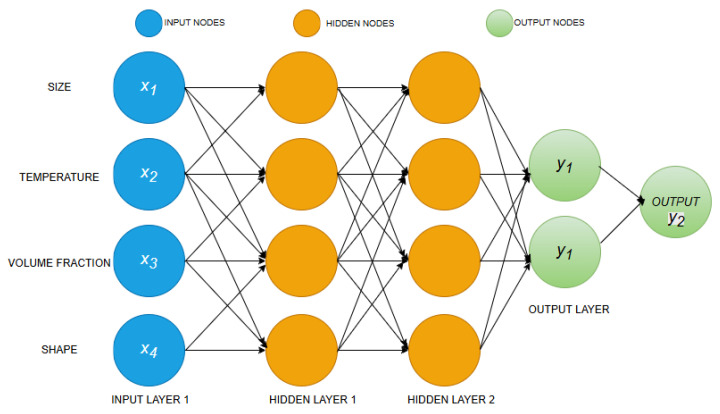
Schematic diagram of an ANN.

An ANN is an information processing model derived from biological nerve systems, such as the brain. It is made up of many interconnected processing units called neurons. Each neuron is connected to the input signals by links, each with a weight. ANNs are widely used to model the thermophysical properties of nanofluids, thereby improving the overall accuracy of these properties. Time and cost can be reduced by using an ANN compared to experimental methods. Y. AbuShanab et al. [44] used an ANN to predict the dynamic viscosity of polyalpha-olefin boron nitride nanofluids. The schematic diagram of an ANN is shown in Figure 2.

### 3.2. Support Vector Machine (SVM)

**Figure 3:**
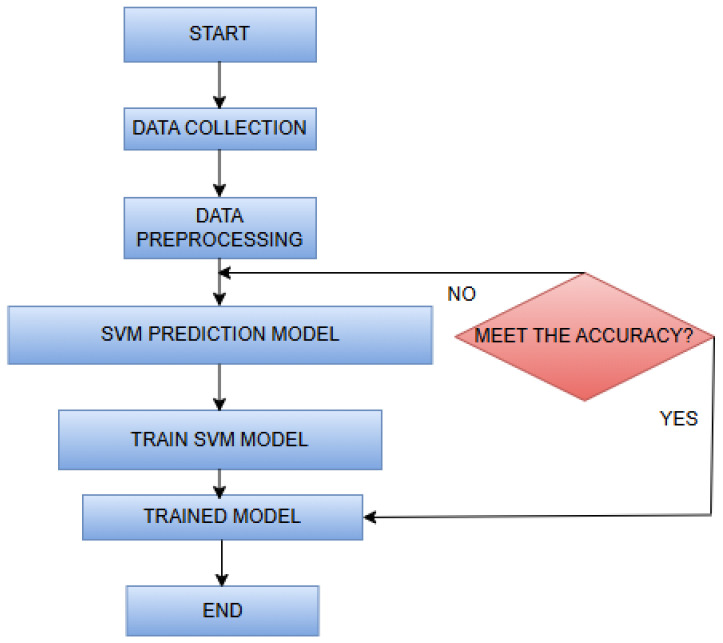
SVM flow chart for prediction model figure recreated based on [63].

Support vector machine (SVM) is a supervised learning technique introduced initially in 1995 for both classification and regression tasks. The method consists of two main formulations: support vector classification and support vector regression (SVR). When used for continuous output prediction, the approach is known as SVR and serves as an effective alternative to conventional regression techniques. SVM-based models have been extensively applied to estimate the thermophysical properties of nanofluids and hybrid nanofluids. For example, Amin Asadi et al. [63] utilized this approach to predict the thermophysical properties of MWCNT/ZnO–engine oil hybrid nanofluids. The schematic representation of the SVM methodology is illustrated in Figure 3.

### 3.3. Multilayer Perception (MLP)

**Figure 4:**
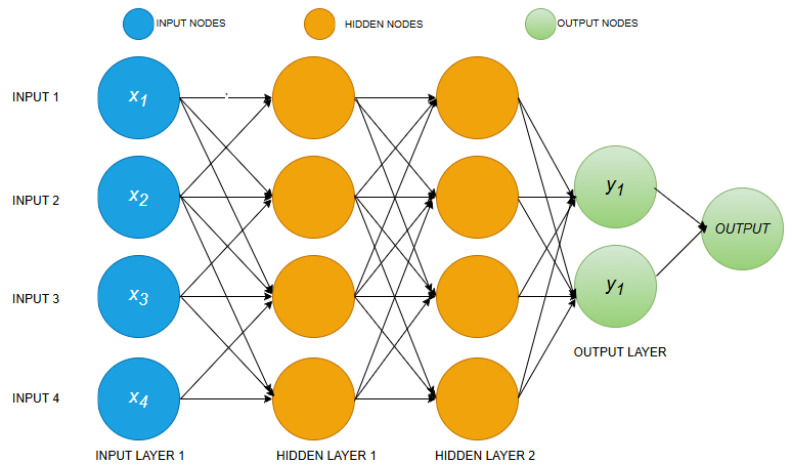
Schematic diagram of MLP.

A multilayer perceptron (MLP) is a supervised learning algorithm that uses multiple layers of neurons between the input and output layers, called hidden layers. This is one of the most commonly used machine learning methods because it can learn nonlinear relationships in data. Given the problem’s complexity, this study considers the number of hidden layers. A schematic diagram of MLP is shown in Figure 4.

## 4. Recent Models to Find the Viscosity, Thermal Conductivity, and Specific Heat of Nanofluids

### 4.1. Thermal Conductivity Models

**Figure 5:**
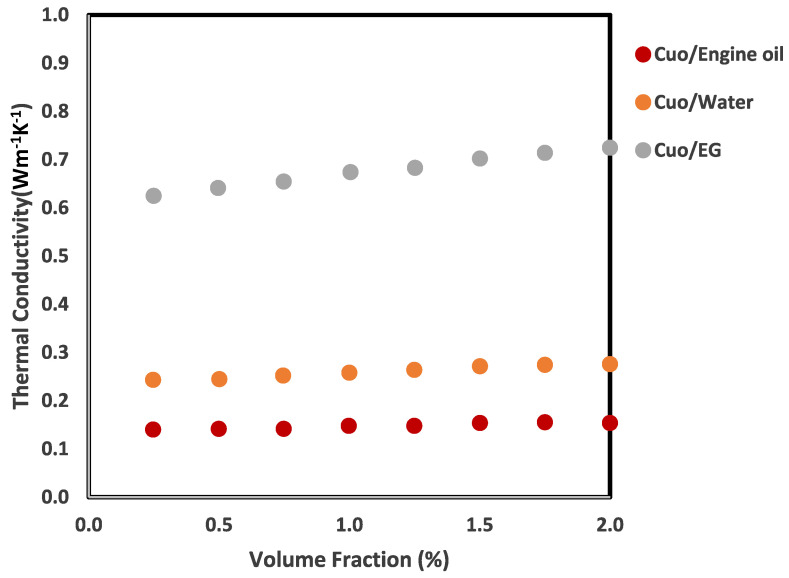
TC of the nanofluids at 10 °C using different base fluids and volume fractions; figure recreated based on [11].

**Figure 6:**
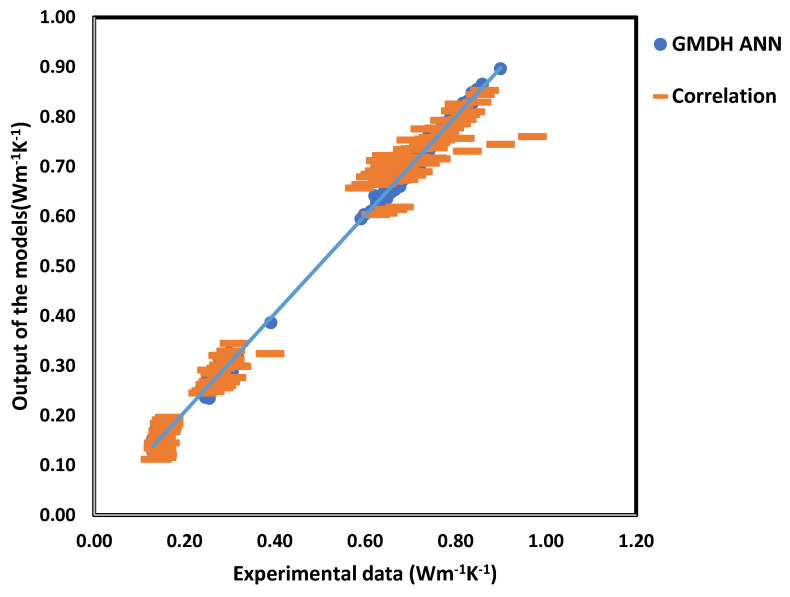
Prediction obtained from GMDH ANN algorithm; figure recreated based on [11].

**Figure 7:**
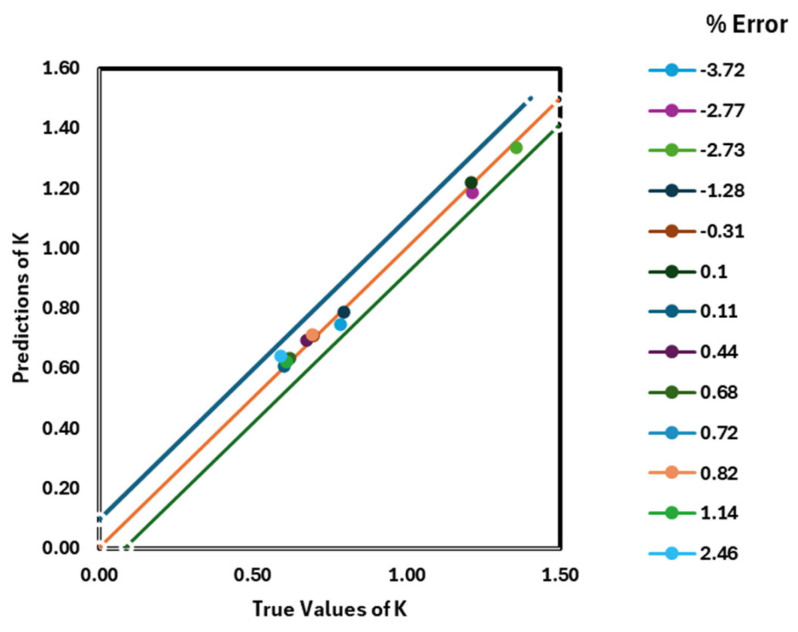
Predictions obtained using random forest regressor; figure recreated based on [16].

Thermal conductivity is a thermophysical property that characterizes the rate at which heat is conducted through a material due to a temperature gradient. Because metals have free electrons that can move freely, they have higher thermal conductivity. An essential characteristic used to assess a fluid’s heat transport capacity is its thermal conductivity. Because of their exceptional thermal conductivity, nanofluids have attracted rapid interest across a wide range of sectors. The working fluid’s increased thermal conductivity will enhance heat transfer. According to a paper from the Argonne National Laboratory, nanofluid thermal conductivity is influenced by eight characteristics, based on approximately 124 research experiments. They are the proportions of particle volume, particle-based materials, particle size, particle form, foundational fluid substance, temperature, intensity, and acidity. Both heat transfer characteristics and thermal performance improve as thermal conductivity increases. There are numerous benefits of using nanofluids in heat transfer, including lower radiation levels and smaller heat exchangers with higher heat transfer efficiency. By utilizing nanofluids in the pumping process, more energy can be conserved and used for heat transfer, enabling estimation of the heat transfer coefficient, prediction of thermal loads, assessment of cooling energy consumption, and accounting for solar radiation. Thermal conductivity is a valuable property for heat transfer in various applications, including solar and geothermal energy sources. To predict the thermal conductivity of nanofluids containing CuO nanoparticles, Ali Komeilibirjandi et al. [11] proposed a group-based data management approach and mathematical relations that account for the base fluid thermal conductivity, temperature, nanoparticle concentration, and nanoparticle diameter. A set of neurons forms the multilayered, feedforward network structure of the GMDH neural network. By limiting divergence, this design connects the network’s structure and form. They utilized the following polynomials for the evaluation.

(1) $$R^{2}=1-\frac{\sum_{i=1}^{i=n}({y_{i}^{experimental}-y_{i}^{predicted})}^{2}}{\sum_{i-1}^{i=n}{\left(y_{i}^{experimental}-y^{experimental}\right)}^{2}}$$

(2) $$RD_{i}=\frac{y_{i}^{experimental}-y_{i}^{predicted}}{y_{i}^{experimental}}\times 100$$

(3) $$\mathrm{MSE}=\frac{1}{n}\sum_{i=1}^{i=n}{\left(y_{i}^{experimental}-y_{i}^{predicted}\right)}^{2}$$

(4) $$\mathrm{AARD}=\frac{1}{n}\sum_{i=1}^{i=n}\left|\frac{y_{i}^{experimental}-y_{i}^{predicted}}{y_{i}^{experimental}}\times 100\right|$$

where $y_{i}^{experimental}$, $y_{i}^{predicted}$ and $y^{experimental}$ represent the values obtained from the experimental and model, and the mean value of the experimental, respectively, and yielded an R^2^ value of 0.9996. Here, it is shown that the increase in thermal conductivity is more strongly influenced by parameters such as temperature, concentration, and particle size, as depicted in Figure 5. The obtained MSE values in this work are 2.36 × 10^−5^ and 9.28 × 10^−4^ for the GMDH ANN and the mathematical correlation, respectively. Referring to Figure 6, the GMDH ANN provides greater accuracy than mathematical correlation across all statistical criteria. To forecast the thermal conductivity of the titanium oxide–water nanofluid, P. Sharma et al. [64] created five distinct machine learning techniques (ANN, GBR, SVR, DTR, and RFR) by considering form, size, mass percentage, temperature, and base fluid thermal conductivity, using 228 collected data points. This research demonstrated that nanoparticle shape also affects the predicted thermal conductivity of the nanofluid. Using R^2^ and MSE values, they compared the models and found that the model resulted in a value of R^2^ = 0.98, as shown in Figure 7. Random forest (RF) was the more effective algorithm for estimating the thermal conductivity of the TiO_2_-H_2_O nanofluid. To calculate the thermal conductivity of carbon-based nanoparticles, such as single-walled carbon nanotubes (SWCNTs), MWCNTs, graphite, graphene, and carbon nanotubes (CNTs), M. Taheri et al. [65] developed ANN and RSM (response surface method) models. The developed models used the following inputs: temperature, mass fraction, sizes, thermal conductivities of Phase Change Materials (PCMs) and nanoparticles, and the PCM phase. They used 482 experimental values gathered from diverse sources to create models. Six neurons make up the input layer of the ANN model, eight neurons make up the hidden layer, and a single neuron makes up the output layer. The activation functions in the outer and hidden layers are linear and tanh, respectively.

**Figure 8:**
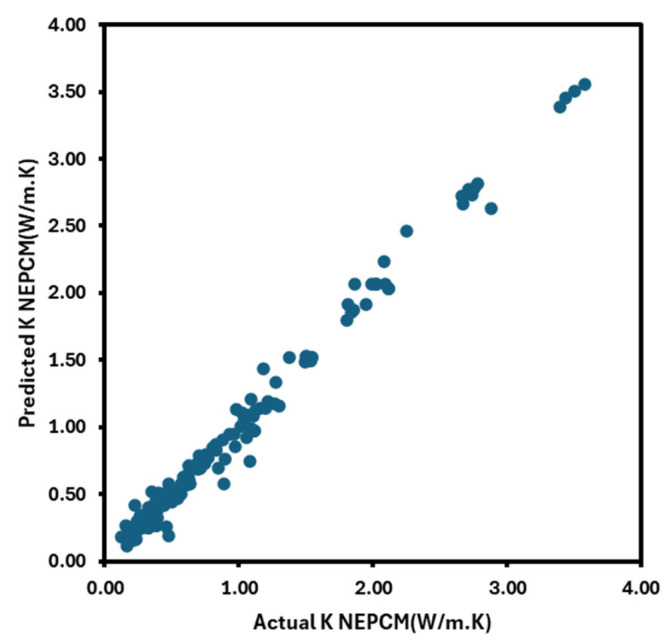
Comparison between the real and estimated values obtained by ANN; figure recreated based on [65].

Additionally, 80% of the data points were used to train the Levenberg–Marquardt backpropagation method, resulting in improved performance, as shown in Figure 8. Response surface methodology (RSM) was used to predict the thermal conductivity of materials undergoing nanophase shift and to develop formulas for both solid and liquid phases. When the R^2^ values of the two models were compared, the ANN performed better than the RSM model. To identify enhanced patterns and contextualize the modeling and comparison analyses described in the following sections, Table 1 provides a summary of the reported thermal conductivity values for several nanofluids.

### 4.2. Viscosity Model

A crucial characteristic in assessing nanofluid performance is viscosity, which quantifies a fluid’s resistance to flow. Viscosity variations affect momentum and energy transport mechanisms, which in turn influence a nanofluid’s thermal capacity and heat transfer efficiency. Factors influencing the nanofluid’s viscosity include base fluid type, particle size, particle form, volume concentration, temperature, pH value, shear rate, surfactants, preparation method, and particle aggregation. Viscosity significantly affects flow parameters, including the Reynolds number, heat transfer coefficients, and pressure drop. Viscosity is most important in systems that need flow. The greater the resistance, the higher the viscosity, which fluctuates with temperature.

Hydraulic braking systems use nanofluids due to their high viscosity. Ilia Chiniforooshan Esfahani [66] developed a technique that uses a Multi-Fidelity Neural Network (MFNN) and incorporates physical principles into the model to estimate nanofluid viscosity. The diameter, density, temperature, and volume percentage of the nanoparticles were used as inputs when developing the model, which was built using 1425 experimental data points: 30% are used for testing and 70% for training.

**Figure 9:**
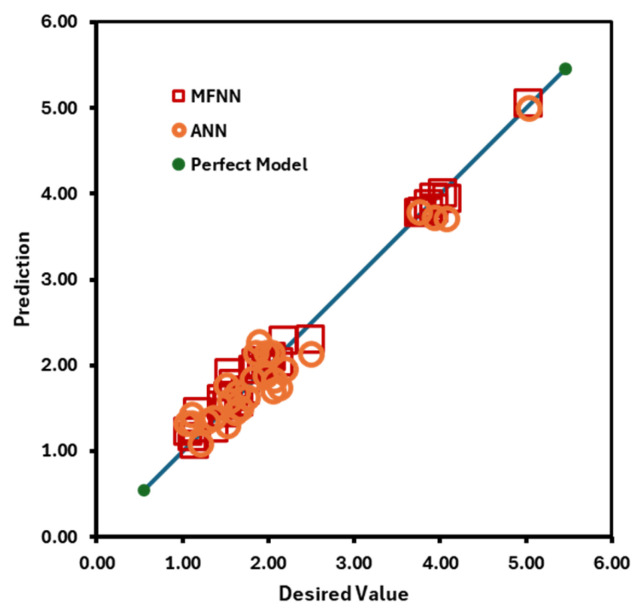
Comparison of MFNN and theoretical model; figure recreated based on [66].

**Figure 10:**
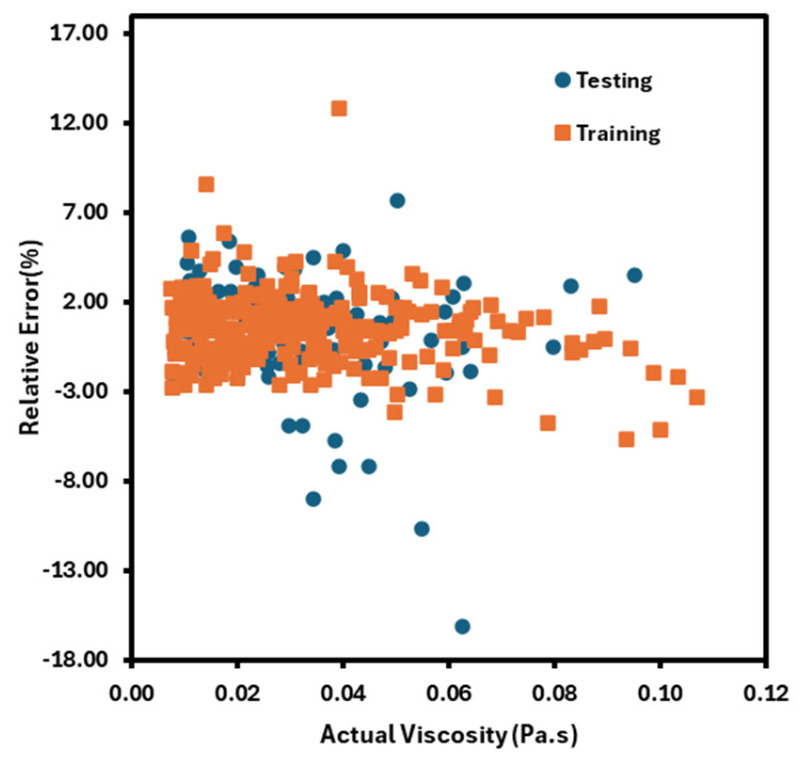
Relative error of RF for the prediction of viscosity; figure recreated based on [67].

Nonlinear activation functions, including ReLU and tanh, are used here. When the MFNN’s performance was compared to theoretical models, as illustrated in Figure 9, the MFNN performed best with two hidden layers, each with 13 and 18 neurons, yielding an R^2^ value of 0.991. Random forests (RF), an artificial intelligence technique, were employed by Kai Xiang Tan et al. [67] to find the viscosity of an alumina-based nanofluid. Here, temperature, volume concentration, and shear rate are taken as input variables. Then, the RF model is implemented in Python 3.9, and the nanofluid viscosity is estimated using the RF regressor approach, with an 80-20 split for training and testing. Checking R^2^, A-R^2^, MSE, RMSE, and MAE allows for the evaluation of the model’s performance using the following formula. There are n data points and p independent variables: N = total data points in the dataset; $y_{i}\;$= actual values from the dataset; and = predicted values from RF. The calculated value was close to one, and the maximum relative error percentage ranged from 12.657% to −16.071%, as shown in Figure 10.

(5) $$R^{2}=1-\frac{Sum\;of\;squared\;residual}{Sum\;of\;squared\;total}$$

(6) $${A-R}^{2}=1-\left(1-R^{2}\right)*\frac{n-1}{n-p-1}$$

(7) $$\mathrm{MSE}=\frac{1}{N}\sum_{i=1}^{N}{\left(y-y_{i}\right)}^{2}$$

(8) $$\mathrm{RMSE}=\sqrt{\frac{\sum_{i=1}^{N}{\left(x-x_{i}\right)}^{2}}{N}}$$

(9) $$\mathrm{MAE}=\frac{\sum_{i=1}^{N}\left|y-y_{i}\right|}{n}$$

### 4.3. Specific Heat Models

**Figure 11:**
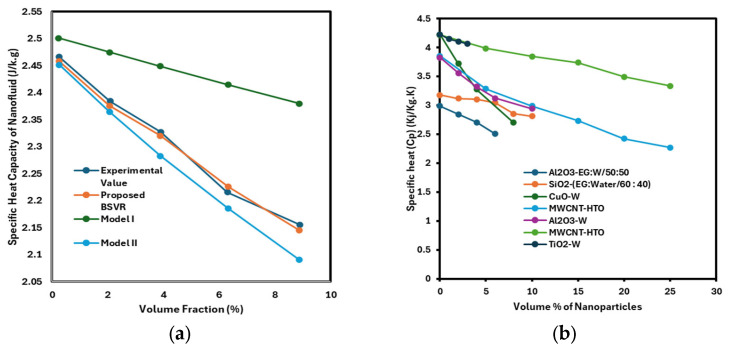
Impact of nanoparticle volume fraction on the specific heat capacity using different models (**a**) and of different nanofluids (**b**), figure recreated based on [68,69].

Specific heat is defined as the quantity of heat required to raise the temperature of a substance by a unit amount. It varies with the base fluid mode and decreases as intensity increases. The inclusion of nanoparticles at different medium temperatures alters the specific heat of the base fluids. This is positively impacted by temperature. Reliable studies reported that the nanofluid-specific heat declined moderately as the particle volume fraction increased. For engineers working in fluid flow management and heat transfer, the specific heat of nanofluids is crucial. The heat exchanger’s energy balances will be assessed using specific heat. Using 84 experimental datasets, Ibrahim Olanrewaju Alade et al. [39] developed a Bayesian support vector regression (BSVR) model to forecast the specific heat capacity of Al_2_O_3_–ethylene glycol nanofluid. By calculating the RMSE, MAE, AARD, and R2, the accuracy was examined. The specific heat capacity reduced by increasing the volume percentage in this investigation, as shown in Figure 11a.

## 5. Machine Learning Models to Estimate Nanofluid Thermal Conductivity and Viscosity

Machine learning uses algorithms tailored to a given task. The volume of input data affects how well these algorithms function. This is not the analytical study of models, but rather the analysis of dynamical systems that depend on the observed facts. It is challenging to design models for dynamic systems like heat transfer, fluid flow, climate dynamics, biological systems, and brain dynamics. Machine learning focuses on algorithms that improve performance as more data is added. The thermophysical characteristics of nanofluids can be predicted using these techniques. Nanofluids’ thermophysical characteristics are predicted here using a variety of machine learning techniques, including multilayer perceptron, SVM, and ANN. Research indicates that ANNs are more accurate at predicting the thermophysical properties of these fluids. Below is a discussion of a few models. Figure 11b illustrates the effect of nanoparticle volume fraction on the thermal conductivity of various nanofluid compositions. The results indicate that, across all nanofluids considered, the specific heat capacity decreases with increasing nanoparticle volume fraction.

### 5.1. Thermal Conductivity Prediction Models

An ANN was developed by Mohammad Hemmat Esfe et al. [9] utilizing seventy-two experimental data points to forecast the thermal conductivity of the ferrous–ethylene glycol nanofluid. When building the model, the solid volume percentage, temperature, and particle size were selected as inputs. A total of 70 percent of the 72 experimental data points were used for training, 15 percent for validation, and 15 percent for testing. There were five neurons in the second hidden layer and seven neurons in the first hidden layer of the ANN. The MSE was 0.00016, and the largest difference between the estimated and experimental values was 2%. This fluid finds uses in the nuclear, automotive, and electronics industries. This study showed excellent agreement with the presented correlation and experimental data. Moreover, this study used only temperature and solid volume fraction as input variables to predict thermal conductivity, without accounting for other factors that affect this property. By using the diameter, volume fraction, and temperature of the nanofluid, and TC of the base fluid as inputs, Shankar Durgam et al. [10] created a model. They predicted the thermal conductivity of nanofluids such as Al_2_O_3_-H_2_O, Al_2_O_3_-EG, CuO-H_2_O, CuO-EG, TiO_2_-H_2_O, and TiO_2_-EG using 473 experimental data points; 30% of the data collected was used for testing, and the remaining 70% for training. ANN models help cut expenses and time. Moreover, at temperatures above 25 °C, the investigational thermal conductivity data is not extremely close to anticipated analytical and ML model values. To forecast the thermal conductivity of CuO nanoparticles using base fluids such as EG, water, and motor oil, Ali Komeilibirjandi et al. [11] developed a neural network with input variables including the base fluid thermal conductivities, concentration, temperature, and nanoparticle diameter. This was developed by utilizing a genetic algorithm. In this case, the AARD value is 0.881%, and the ANN coefficient of correlation is 0.9996. Mohammad Hemmat Esfe et al. [12] utilized an ANN model to estimate the thermal conductivity of Mg (OH)_2_–EG using volume concentration and temperature as inputs. A total of 15% of the dataset was used for validation, 15% for testing, and 70% for training. The absolute maximum error was 1.50 × 10^−3^. This work showed that ANNs can be used to reduce cost and time. Using 776 experimental data points, E. Ahmaddloo and S. Azizi [2] built an ANN to estimate the thermal conductivity of 15 nanofluids. For training, validation, and testing, respectively, 544, 116 and 116 data points were utilized. The model’s inputs included the average nanoparticle diameter, volume fraction, thermal conductivity, and temperatures of the nanoparticles and the base fluids. They used a Feedforward Multilayer Perception (MLP) network with a single hidden layer, training by the Levenberg–Marquardt backpropagation technique, and transfer function as linear and tangent sigmoid. The network was run 50 times each, with a range of 1 to 23 neurons. After analyzing 18 neurons, the optimal outcome was discovered with a correlation coefficient of 0.994 and an MAPE of 1.31%. These are time-saving and cost-effective methods for estimating the thermal conductivity of nanofluids. This work also showed good fitting between experimental and predicted values. An ANN model was designed by Mohammad Hemmat Esfe et al. [13] to study the thermal conductivity of an aluminum oxide–water nanofluid by using temperature and solid volume percent as inputs. The volume fractions in this case are 0.0025, 0.005, 0.01, 0.02, 0.03, 0.04, and 0.05%, while the temperature ranges from 26 to 55 degrees Celsius. They utilized 28 data points, dividing it into 70% for testing and 30% for validation. The network’s hidden layer had nine neurons, and the corresponding MSE and correlation coefficient were 2.42 × 10^−6^ and 0.99988, respectively. Because of their improved performance, these models can be utilized to simulate the heat conductivity of an aluminum oxide–water nanofluid. This work suggested that correlation and ANN models can be applied to predict the thermal conductivity of H_2_O-based nanofluids; moreover, ANN performance is better than that of the correlation model. Considering temperature, volume concentration, and nanoparticle size as inputs, Kumar and Kavitha [29] employed Gaussian process regression (GPR) to predict the thermal conductivity of an Al_2_O_3_–water nanofluid in MATLAB. They obtained an R-square value of 0.99. This work clearly stated that temperature is one of the most influential factors in predicting the thermal conductivity of nanofluids. The thermal conductivity of nanofluids containing gamma-Al_2_O_3_, TiO_2_, and CuO with Carboxymethyl Cellulose (CMC) is predicted by Shateri et al. [17] using temperature, nanoparticle concentration, and nanoparticle thermal conductivity as inputs. They noticed that thermal conductivity increases with an increase in both temperature and concentration. In this instance, the temperature ranges from 5° to 45° Celsius, with volume concentrations of 0.1%, 0.2%, 0.5%, 1.5%, 3.0%, and 4.0%. The sigmoid function is used as the transfer function in a three-layer feedforward neural network, yielding an average error of 1.6% and a maximum error of 5.8%. This work showed that variation in volume fraction is the most affecting parameter on the relative viscosity of nanofluids. Kurt et al. [16] used 40 experimental data points, 32 for training and 8 for testing, to develop a model to estimate the thermal conductivity of an ethylene glycol–water solution as a function of experimentally determined variables. The model considered temperature, concentration, and density. In the proposed artificial neural network architecture, the 3-4-1 design used the sigmoid function as the transfer function. The developed model showed R^2^ value in the range of 0.9999 and MAPE in the range of 0.7984%. This paper proved that ANNs can be used to predict the thermal conductivity of ethylene glycol–water solutions. Using temperature, volume fraction, and nanoparticle size as inputs, Elsheikh et al. [22] created Self-Organizing Maps (SOMs), an unsupervised ANN, to forecast the thermal conductivity of nanofluids containing water, aluminum oxide, copper oxide, and titanium oxide. The designed model was trained using both theoretical and experimental data. The diameter ranges from 5 to 100 nm, the volume fractions are 3.2, 5.8, and 8.4%, the temperatures are 26.6, 30.2, and 33.8 °C, and the MSE and MAPE are 8.2908 × 10^−5^ and 0.5978%, respectively. This work can be used to predict other thermophysical properties of nanofluids. Yashawantha and Vinod [24] developed an ANN model to predict the thermal conductivity of fluids, such as CuO, Al_2_O_3_, and TiO_2_, in EG–water at temperatures ranging from 30 to 60 °C and volume fractions of 0.2 to 2%. In MATLAB, the Levenberg–Marquardt learning algorithm was used for learning, and the neural network tool was used for modeling. Tan-sigmoid and pure line neural transfer functions, respectively, were employed for the hidden layer and output layer. In this instance, 75% of the collected data was used for testing and 25% for training, yielding an R^2^ value of 0.692. The model can be further improved by accounting for additional parameters that affect nanofluid thermal conductivity. Using temperature, volume fraction, the molar mass of base fluids, critical pressure, temperature, acentric factor, and thermophysical parameters of base fluids, Mirsaeidi and Yousefi [28] built an ANN model to predict the thermal conductivity of carbon quantum dots. About 75% of the data collected was used for training, 15% for testing, and 15% for validation. This neural network’s R^2^ value in MATLAB was 0.99867. This work showed that thermal conductivity increases with the addition of nanoparticles to the fluid. Based on particle size, temperature, and volumetric concentration, Ahamadi et al. [38] developed an ANN model to predict the thermal conductivity of an Al_2_O_3_/water nanofluid, achieving a coefficient of correlation of 0.87575, which can be further improved.

### 5.2. Viscosity Prediction Models

With temperature, base fluid viscosity, volume concentration, size, and density as inputs, Shateri et al. [17] built an ANN model to study the viscosity of nanofluids. Using 3144 experimental data points, the proposed eight models were trained on 85% and tested on the remaining 15%. The average absolute relative error of the MLP network with two hidden layers and a tan-sigmoid transfer function was 4.931%. This work showed that MLP performance was comparable to that of the other models. Temperature, volume concentration, and shear rate were used as inputs when Esfe et al. [18] created an ANN to forecast the viscosity of an Al_2_O_3_-MWCNT/5W50 hybrid nano-lubricant. They utilized 204 trial datasets, with 70% allocated to training, 15% to testing, and 15% to validation. For training, Levenberg–Marquardt (LM) backpropagation was employed. Temperature ranged from 5° to 55° Celsius, volume fraction from 0% to 1%, and shear rate from 666 to 10,664. The second layer used f (x) = x as a transfer function, whereas the input layer used the logistic function—f(x) = 2/(1 + e^−x^) and tan sigmoid—f(x) = tanh(x) =2/((1 + e^−2x^) − 1) as transfer functions. R^2^ (correlation coefficient), MAPE (mean absolute percentage error), and MSE (mean squared error) were used to assess networks. The ANN model’s statistical parameters were MAPE = 0.07, R^2^ = 0.998, and MSE = 5.1. In this, an ANN was developed using shear rate, temperature, and volume fraction as inputs. To forecast viscosity, Heidari et al. [19] created an MLP-ANN that considers the following inputs: temperature, base fluid viscosity, volume concentration, density, and nanoparticle diameter, and split the 1490 experimental data into 90% for training and 10% for testing. The network in this instance comprises 25 and 14 neurons in two hidden layers each, and the Levenberg–Marquardt algorithm was applied. For the hidden and output layers, the logistic sigmoid and purelin were chosen as the transfer functions; the corresponding AARD and R^2^ values were 0.4998 and 0.41%, respectively. They developed an ANN model to predict the viscosity of nanofluids with a wide range of operating parameters. Ahmadi et al. [20] created different machine learning models that use temperature, concentration, and nanostructure size as inputs to forecast the viscosity of a copper oxide–water nanofluid. The models developed include MARS, MPR, ANN-MLP, GMDH, and M5-trees. The employed approach was least-squares multivariable polynomial regression (MPR) to determine the best fit. This model has an R^2^ value of 0.9938 and a temperature range of 10 to 66 degrees Celsius, along with size and concentration variations of 11 to 29 nm and 0 to 9%, respectively. The ideal basis functions were determined using the MARS model. It has sixteen basis functions, six of which are independent, and an R^2^ value of 0.9953. The Levenberg–Marquardt method is used in the ANN-MLP model due to its high accuracy and speed. Of the whole dataset, 25% was used for testing, 15% for validation, and 75% for training. Tanh and identity functions are utilized in this network to activate the hidden and output layers, and the R^2^ value obtained is 0.9997. The self-organizing GDMH network reduces the square error between the real and predicted values, yielding an R^2^ score of 0.9994. The M5 tree models are derived from regression models that include linear functions, as well as nodes, branches, leaves, and roots. The R^2^ value of these models is 0.9938. This work concluded that concentration has a greater influence on the prediction of the nanofluid’s dynamic viscosity than nanoparticle size. Using temperature, volume percentage, diameter, particle size, and base fluid parameters as inputs, Longo et al. [21] built an ANN model to estimate the oxide nanoparticle viscosity in EG and water. Thirty collected data points were divided into three categories: training (75%), validation (15%), and testing (15%). The nanoparticle dimension in the network was set at 15 nm, and the temperature and volume fraction were set at 1, 10, 20, 30, and 40 °C, and 1, 2, and 4%, respectively. In this network, the Levenberg–Marquardt algorithm was used with nine neurons, hyperbolic functions as transfer functions for the hidden layer, and linear functions for the output, yielding a mean absolute percentage error of 1.91%. This result will be useful to study the stability of nanofluids. Gaussian process regression (GPR) was used by Kumar et al. [29] to construct an ANN model that predicts the viscosity ratio of Al_2_O_3_–water. After comparing the predicted data with 222 experimental data points, the temperature, volume percent, and nanoparticle diameter were used as inputs. An R^2^ value of 0.09 and an RMSE of 0.000045 were obtained. This paper shows that the exponential kernel function provides accurate predictions of dynamic viscosity. Olumegabon et al. [27] used 120 experimental data points, including temperature, volume fraction, and diesel oil viscosity, as inputs to train machine learning models to predict the viscosity of carbon-based nanomaterials dispersed in oil using a support vector regression model. Using the SVR technique and the Bayesian algorithm, 80% of the data was used for training and 20% for testing in the Math Lab. The results showed a correlation coefficient of 0.9998 and an RMSE of 0.0076 Pa s. This study showed that diesel oil viscosity is the primary predictor of carbon-based nanomaterial viscosity. To forecast the viscosity of carbon quantum dot nanofluids at different temperatures and mass fractions, Mirsaeidi et al. [28] developed an ANN model. Relative viscosity was shown to increase in this investigation as the volume fraction increased, yielding an AAD of 1.29% and an R^2^ of 0.99994 with the experimental data. This result showed that viscosity increases by increasing the volume fraction and decreases with increasing temperature. Using 1490 experimental data points, Harooni et al. [35] created a radial basis function network to estimate the viscosity of Al_2_O_3_, TiO_2_, CuO, and SiO_2_ with various base fluids. This work shows that the volume fraction of nanoparticles has the greatest impact on viscosity, while temperature has the least. Ahmadi et al. [38] develop models based on particle swarm optimization–artificial neural networks (PSO-ANNs) and genetic algorithm–least square support vector machines (GA-LSSVMs) to predict the dynamic viscosity of Fe_2_O_3_/EG–water using temperature, volume fraction, and the mass ratio of EG/water as inputs. This study used feedforward artificial neural networks (ANNs) with one hidden layer, linear or nonlinear activation functions, and the PSO algorithm. The data points were split in an 80-20 ratio. The R^2^ and MSE values were compared. For training and testing, 126 and 100 data points were used for LSSVM models, which produced R^2^ values greater than 0.998 and MSE values less than 0.5. This outcome demonstrated that the GA-LSSVM outperforms the PS0-ANN in prediction accuracy. This study showed that the mass ratio of EG to water has the greatest impact on dynamic viscosity. Using 1620 experimental data points, Ansari et al. [40] constructed ANN models to predict nanofluid dynamic viscosity with different base fluids. To build a network, they used inputs such as temperature, shear rate, nanoparticle concentration, size, and density. The network has a single hidden layer and twenty-three neurons. The output and hidden layers use purelin and tanh as transfer functions. Using 70% of the data for training, it was concluded that the Levenberg–Marquardt algorithm produced the best results among the training algorithms, with an R^2^ value of 0.9954 and an MSE of 0.00901. This study investigated the ability of feedforward backpropagation to estimate dynamic viscosity for various nanofluids. To predict TiO_2_ nanotube dynamic viscosity in ethylene glycol–water-based nanofluids, Ali et al. [41] constructed an ANN model that took temperature, shear rate, and nanotube concentration into account. Using 30% of the data for testing and validation, and the remaining 70% for training, it was found that the mass fraction of TiO_2_ nanotubes is less relevant than the temperature’s impact on dynamic viscosity. Their ANN produced excellent accuracy, with an R^2^ value of 99.99% and an Average Absolute Deviation (AAD) of 0.1981%. In this study, it is observed that temperature affects dynamic viscosity compared to the mass fraction of TiO_2_ nanotubes. To predict the viscosity of carboxylic diamond and multiwalled carbon nanotube nanoparticles scattered in water, A.A.A.A. Alrashed et al. [42] developed ANFIS and ANN models, with volume fraction, temperature, and particle type as input variables. They demonstrated that an ANN is the best model for estimating the thermophysical properties of nanofluids and evaluated it using MAPE and RMSE. To estimate the dynamic viscosity of nanofluids containing MgO nanoparticles, Fadhl et al. [47] developed two intelligent approaches, GMDH and ANFIS. To study the viscosity of thermal oil and multiwalled carbon nanotubes, Maqsood et al. [48] developed ANN and RSM models that accounted for temperature and nanoparticle concentration. The degree of freedom, mean squared, and the sum of squared deviations of the model were determined for each input using an ANOVA optimization technique. Table 2 shows the features of thermophysics in prior research and implementations. Table 3 summarizes the key correlation equations used to calculate the viscosity and TC of nanofluids.

Table 4 presents a comparative analysis of ML models for nanofluid property prediction. For highly nonlinear parameters such as viscosity and thermal conductivity, ANN and ensemble models often outperform simpler ML techniques. SVR models perform exceptionally well for smoothly varying quantities, such as specific heat capacity and density. Because hybrid nanofluids are more complex, larger datasets and more sophisticated machine learning models are needed. No single ML model is universally optimal; model applicability depends on property type, nanofluid composition, and data quality.

## 6. Summary

This work reviews research on modeling the viscosity, specific heat, and thermal conductivity of nanofluids. These studies indicate that thermal conductivity increases with temperature and volume fraction, whereas viscosity decreases with size, temperature, and volume fraction. Based on the critical synthesis of experimental and machine learning-based studies reviewed in this work, the following key conclusions are drawn:

1. Machine learning models consistently outperform classical theoretical and semi-empirical correlations in predicting nanofluid viscosity and thermal conductivity, primarily because they can capture strong nonlinear interactions among temperature, nanoparticle volume fraction, particle size, and base fluid properties.
2. Among ML techniques, artificial neural networks (ANNs) and ensemble learning approaches demonstrate superior performance for highly nonlinear properties such as viscosity and thermal conductivity, particularly in oxide, carbon-based, and hybrid nanofluids, where traditional correlations exhibit significant deviations.
3. In contrast, support vector-based models achieve comparable accuracy for more smoothly varying properties, such as density and specific heat capacity, suggesting that simpler ML architectures may be adequate when nonlinear effects are less pronounced.
4. The predictive accuracy and robustness of ML models are strongly dependent on the quality, diversity, and operating range of the training datasets, highlighting that models trained on narrow temperature or concentration ranges exhibit limited generalization.
5. Temperature and nanoparticle volume fraction are consistently identified as the most influential input features for both viscosity and thermal conductivity prediction. At the same time, particle size and shape play secondary but non-negligible roles, particularly in hybrid and non-spherical nanoparticle systems.
6. ANN architecture selection—including the number of hidden layers, neurons, and activation functions—significantly affects model performance, and inadequate tuning often leads to overfitting or unstable predictions despite high training accuracy.
7. Although ML models demonstrate excellent interpolation within their trained parameter spaces, their extrapolation performance remains limited, underscoring the need for rigorous validation, uncertainty quantification, and physical consistency checks.
8. The integration of experimental measurements with data-driven modeling provides a reliable, computationally efficient framework that significantly reduces reliance on extensive experimental campaigns while maintaining high predictive accuracy.
9. Model transferability across different nanofluids remains limited, as most ML models are trained on specific nanoparticle-based fluid combinations, underscoring the need for broader, standardized datasets.
10. Machine learning-based optimization frameworks offer strong potential for identifying optimal nanoparticle types, sizes, and concentrations for application-specific requirements, such as data center cooling and high-heat-flux electronic thermal management.
11. To improve generalization, interpretability, and extrapolation, future ML models should incorporate physics-informed constraints that link conservation laws and thermodynamic principles to data-driven learning.
12. Finally, the development of unified machine learning frameworks capable of simultaneously predicting multiple thermophysical properties (density, specific heat, viscosity, and thermal conductivity) is identified as a critical step toward accelerating the practical deployment of nanofluids in real-world thermal systems.

## Figures and Tables

**Table 1:** Nanofluid thermal conductivity.

**Metalic** **Solids**	**TC** $\left(Wm^{-1}K^{-1}\right)$	**Non Metalic** **Solids**	**TC** $\left(Wm^{-1}K^{-1}\right)$	**Liquids**	**TC** $\left(Wm^{-1}K^{-1}\right)$
Aluminum	237	Beryllium ox-ide (BeO)	40	EG	0.20
Copper	401	Carbon nanotubes	3000	Engine oil	0.14
Gold	318	CuO	76.50	Glycol	0.29
Silver	429	Diamond	3300	Water	0.6
		Silicon	148		

**Table 2:** Features of thermophysics of prior research and implementations.

Nano Particle/Base Fluid	Input Parameters	Model	Property	Source	Remarks	Comparative Insight
Al_2_O_3_/Water	Nanoparticle volume fraction, temperature	ANN	TC &Viscosity	[7]	An increase in nanoparticle concentration and fluid temperature can lead to higher thermal conductivity and viscosity.	Demonstrated that the ANN provides a useful method to predict thermophysical properties of nanofluids with limited experimental data.
Al_2_O_3_/Water	Temperature, diameter of particle, solid volume fraction	ANN	Viscosity	[9]	In this study, the proposed correlation was validated by comparison with experimental data.	ANN can effectively capture nonlinear relationships between input parameters and thermophysical properties of ferromagnetic nanofluids, achieving high prediction accuracy.
CeO_2_/EG	Fluid viscosity, volume concentration, diameter, molecular weight, temperature	ANN	Viscosity	[10]	Showed that only the ANN model predicts the viscosity value close to the experimental value.	ANN may help lower the expenses, time, and nanofluids associated with traditional experimental methods.
Al_2_O_3_/Water	Average diameter, volume fraction, temperature	ANN	TC	[2]	Proposed ANN model to predict the TC percentage with acceptable accuracy.	Demonstrates the ANN’s capability across multiple nanofluids for thermal conductivity prediction, but does not address viscosity.
Al_2_O_3_/EG/Water	Volume fraction, size and density of nanoparticles, temperature, viscosity of base fluids	MLP, DT, SVR, RF, and ET	Viscosity	[17]	Used an ML model built on the arrangement of 7 baselines, named the committee intelligent system.	Strong multi-model comparison for viscosity prediction, but no other thermal properties.
CuO/Water	Size, temperature, concentration	GMDH	Viscosity	[31]	Mentioned the size of the nanoparticles to be considered to get a precise model.	Combines stability assessment, experiments and ANN but excludes particle size effects.
Al_2_O_3_/EG/Water	Volume fraction, temperature	ANN	Thermal conductivity & viscosity	[5]	The results indicated that the ANN model predicts the properties more accurately than the proposed correlation.	ANN may help lower the expenses, time and nanofluids associated with traditional experimental methods.
Al_2_O_3_/EG, CuO/EG, TiO_2_/Water	Thermal conductivity nanoparticles and base fluids, volume concentration, diameter of nanoparticles, temperature	ANN	Thermal conductivity & viscosity	[10]	The thermal conductivity values predicted by ANN and linear regression matched very well with the experimental results.	ANN may help lower the expenses, time and nanofluids associated with traditional experimental methods.
Al_2_O_3_, CuO, TiO_2_/CMC	Temperature, nanoparticle concentration, thermal conductivity of the nanoparticles	ANN and HC (Hamilton crosser)	Thermal conductivity	[15]	For volume concentrations of 3% and 4%, the thermal conductivities of CuO and TiO_2_ were higher than that of the base fluid.	Provided experimental data and ANN modeling for the thermal conductivity of non-Newtonian nanofluids.
CuO/Oil/EG, Al_2_O_3_/Oil, Mg(OH)_2_/EG, TiO_2_/Water	Average diameter, volume fraction, temperature	ANN	Thermal conductivity	[2]	It was determined that an ANN with a 5-18-1 neuron configuration provides the optimal topology for developing the model.	Provided experimental data and ANN modeling for the thermal conductivity of non-Newtonian nanofluid, but does not predict viscosity.
ZrO_2_/EG	Temperature, concentration	ANN	Viscosity, thermal conductivity, density, specific heat	[46]	The developed model ANN and its correlation analysis perfectly agree with experimental data.	Combines experiments, ANFIS modeling, and new correlations for ZrO_2_/EG nanofluid properties, but excludes particle size effects.
CQD’S/Water/EG	Temperature, volume fraction	ANN	Thermal conductivity, density & viscosity	[28]	Three ANN models to guess TC, density, and viscosity.	Provides experimental data and new correlations, but excludes the particle size effects.
Fe/EG	Temperature, volume fraction	ANN	Thermal conductivity & viscosity	[12]	The proposed correlation showed good agreement with the experimental data.	Demonstrates the superiority of ANNs over empirical correlations and does not compare with more sophisticated machine learning models or particle size features.

**Table 3:** Correlation equations for the thermophysical properties of nanofluids reported in prior studies.

Property	Correlation Equation	Conditions	Source
Thermal Conductivity	$k_{nf}=k_{w}(0.9808+0.0142\emptyset +0.003883T_{b}-0.00068d_{p})$	For 0 ≤ ∅ ≤ 3.7, 20 ≤ *T_b_* ≤ 70, 20 ≤ *d_p_* ≤ 150	[70]
Viscosity	$\mu_{nf}=\mu_{w}(0.9042+0.1245\emptyset -0.00{01206T}_{b}+0.0043d_{p})$		
Thermal conductivity	$k_{nf}\left(1-\emptyset \right)+\beta_{1}k_{p}\emptyset +C_{1}\frac{d_{bf}}{d_{p}}k_{bf}{Re}_{d_{p}}^{2}Pr\emptyset$	$\beta_{1}=0.01,C_{1}=18\times 10^{6},\;d_{bf}=0.384,\;l_{bf}=0.738$	[71]
Viscosity	$\mu_{nf}=\mu_{w}{\left(1+\frac{\emptyset }{100}\right)}^{11.3}{\left(1+\frac{T_{nf}}{70}\right)}^{-0.038}{\left(1+\frac{d_{p}}{170}\right)}^{-0.061}$	$0\leq T_{w}\leq 100,\emptyset <4\%$	[72]
Thermal conductivity	$k_{nf}=k_{w}0.8938(1+\frac{\emptyset }{100}{)}^{1.37}{\left(1+\frac{T_{nf}}{70}\right)}^{0.2777}{\left(1+\frac{d_{p}}{150}\right)}^{-0.0336}(\frac{\alpha_{p}}{\alpha_{w}}{)}^{0.01737}$		
Effective TC	$\frac{k_{eff}}{k_{f}}=1+{4.44Re}^{0.4}{Pr}^{0.66}{\left(\frac{T}{T_{fr}}\right)}^{10}{\left(\frac{k_{p}}{k_{f}}\right)}^{0.03}\emptyset^{0.66}$	294 K≤T≤324 K,0.002≤∅≤0.009	[73]
Effective VC	μeffμf=11−34.87(dpdf)−0.3∅1.03		

**Table 4:** Summary of machine learning models used for predicting nanofluid thermophysical properties.

Source	Thermophysical Properties	Nanofluid Types	ML Models Used	Key Findings/Best Performing Models	Critical Observations & Limitations
[4]	Density	Nitride nanoparticles/ethylene glycol	SVR	SVR showed high accuracy due to smooth and weakly nonlinear density variation	Limited to specific nitride nanofluids; generalization not assessed
[10]	Thermal conductivity & viscosity	Various nanofluids	ANN	ANN outperformed analytical correlations for both properties	Dataset size and preprocessing details limited
[17]	Viscosity	Various nanofluids	ANN	ANN and ensemble models showed superior accuracy	Model interpretability not discussed
[20]	Dynamic viscosity	CuO/water	ANN, SVR, ANFIS, GPR	ANN and ANFIS captured strong nonlinear behavior effectively	Restricted operating range (temperature, volume fraction)
[27]	Viscosity	Carbon-based nanomaterials/diesel oil	ANN, SVR	ANN achieved best performance due to highly nonlinear viscosity trends	No external dataset validation
[36]	Specific heat capacity	Metallic oxides/ethylene glycol	SVR	SVR provided stable and accurate predictions	Focused on limited nanoparticle types
[43]	Multiple properties	Hybrid nanofluids	ANN, ensemble methods	Ensemble models outperformed single learners for hybrid nanofluids	Requires large datasets; data scarcity remains
[44]	Dynamic viscosity	Boron nitride/polyalpha-olefin	ANN, SVR, ANFIS	ANN showed excellent accuracy under wide temperature range	High dependence on training data quality
[45]	Thermal conductivity	Oxide nanofluids	ANN-based models	ANN effectively predicted nonlinear conductivity enhancement	Lacked comparison with ensemble techniques
[57]	Thermal conductivity	Al_2_O_3_/ethylene glycol	ANN, SVR, RF, XGBoost, others	Ensemble and ANN models yielded highest accuracy	Model robustness across datasets not tested
[64,65]	Thermal conductivity	TiO_2_/water	ANN, SVR, GPR	ANN and GPR performed best among tested models	Case-specific study; limited transferability

## Data Availability

Data will be made available upon request.

## Abbreviations

ANN	Artificial Neural Network	ANFIS	Adaptive Neuro-Fuzzy Inference System
SVR	Support Vector Regression	RSM	Response Surface Methodology
MAE	Mean Absolute Error	GMDH	Group Method of Data Handling
RMSE	Root Mean Square Error	MRE	Mean Relative Error
SPM	Single-Phase Model	SHAP	Shapley Additive Explanations
RBF	Radial Basis Function	RF	Random Forest
MAPE	Mean Absolute Percentage Error	RSM	Response Surface Method
RRMSE	Relative Root Mean Square Error	PCM	Phase Change Material
MSE	Mean Squared Error	MFNN	Multi-Fidelity Neural Network
BSVR	Bayesian support vector regression		
SSE	Sum of Squared Errors	SOM	Self-Organizing Map
CMC	Carboxymethyl Cellulose		
LSSVM	Least Square Support Vector Machine		
AARD	Average Absolute Relative Deviation	PS0	Particle Swarm Optimization
GPR	Gaussian Process Regression	AAD	Average Absolute Deviation
GA	Genetic Algorithm	ANOVA	Analysis of Variance
MLP	Multilayer Perceptron		
